# Relationship between haematological data and radiation doses of TEPCO workers before and after the FDNNP accident

**DOI:** 10.1093/jrr/rrac089

**Published:** 2023-01-04

**Authors:** Ryuji Okazaki, Masaoki Kohzaki, Michiaki Kai, Ying Jiang, Tatsuhiko Kubo, Akira Ootsuyama, Toshihiko Sado, Katsunori Suzuki, Seiichiro Tateishi, Koji Mori

**Affiliations:** Department of Radiobiology and Hygiene Management, Institute of Industrial Ecological Sciences, University of Occupational and Environmental Health, Japan, Kitakyushu 807-8555, Japan; Department of Radiobiology and Hygiene Management, Institute of Industrial Ecological Sciences, University of Occupational and Environmental Health, Japan, Kitakyushu 807-8555, Japan; New Department Preparatory Office, Nippon Bunri University, Oita 870-0397, Japan; Department of Health Development, Institute of Industrial Ecological Sciences, University of Occupational and Environmental Health, Japan, Kitakyushu 807-8555, Japan; Department of Public Health and Health Policy, School of Medicine, Hiroshima University, Hiroshima 739-8511, Japan; Department of Radiation Biology and Health, School of Medicine, University of Occupational and Environmental Health, Japan, Kitakyushu 807-8555, Japan; Research Center for Radiation Protection, National Institute of Radiological Sciences, Chiba 263-0024, Japan; Division of Infection Control and Prevention, University of Occupational and Environmental Health, Japan, Kitakyushu 807-8555, Japan; Disaster Occupational Health Center, Institute of Industrial Ecological Sciences, University of Occupational and Environmental Health, Japan, 807-8555, Japan; Department of Occupational Health Practice and Management, Institute of Industrial Ecological Sciences, University of Occupational and Environmental Health, Japan, Kitakyushu 807-8555, Japan

**Keywords:** Fukushima Daiichi Nuclear Power Plant (FDNNP) accident, TEPCO, medical examinations, haematological evidence

## Abstract

We evaluated the correlation between radiation dose and the medical examination data of Tokyo Electric Power Company Holdings, Inc (TEPCO) employees working during the Fukushima Daiichi Nuclear Power Plant (FDNPP) accident in 2011. This study included 2164 male TEPCO workers who received periodic medical examinations from March 2006 to January 2013. First, we conducted log-linear regression analyses using the haematological data of 585 emergency workers and confounding factors to examine the effect of internal radiation exposure in March 2011. Since external radiation exposure was a major influence, we then evaluated the correlation between both internal and external radiation dose and the haematological data of 1801 emergency workers and confounding factors before and after the accident. Among 585 workers, internal radiation exposure in March 2011 alone was mainly due to thyroid doses (0.1-10 Gy) but not to bone marrow (BM) doses (0.01-1 mGy). Compared to before and after the accident, we found that the levels of monocytes, eosinophils (Eos) and basophils increased slightly, whereas the frequency of smoking and alcohol consumption decreased substantially. External dose exposure was positively correlated with haemoglobin (Hb), red blood cell and Eos but negatively correlated with age, haematocrit and frequency of alcohol consumption. Among these variables, Hb exhibited the strongest correlation with external dose. Regarding the correlation with Hb, although there is a possibility that confounding factors other than exposure were not evaluated, our findings on emergency workers can serve as a reference for the evaluation of health conditions during the emergency period of future nuclear-related accidents.

## INTRODUCTION

Large amounts of volatile radionuclides, such as Cs-134, Cs-137 and I-131, were released into the air and the Pacific Ocean after the Fukushima Daiichi Nuclear Power Plant (FDNPP) accident [1–3]. The ambient dose rate caused by the radioactive contamination of debris and rubble followed by hydrogen explosions produced ‘hot spots’ and made the prevention of unwanted internal exposure to volatile radionuclides during emergency work difficult [1, 2, 4, 5]. In the emergency period in March 2011, restoring the cooling system with a stable electrical supply to prevent the risk of reactor explosion and then stabilizing the reactors and decontaminating the water were the most important tasks for emergency workers [6]. Nevertheless, the total amounts of I-131 and Cs-137 emitted into the atmosphere from 12 March through 6 April 2011, were 1.5 × 10^17^ and 1.3 × 10^16^ Bq, respectively. Therefore, the FDNPP accident was also considered an International Nuclear and Radiological Event Scale (INES) level 7 nuclear disaster, and caution dictates the evaluation of internal radiation exposure in thousands of emergency workers in the FDNPP accident [6]. In fact, 133 workers were exposed to more than 100 mSv of radiation in March 2011, although this was changed to 138 workers after a revision in July 2011 [7, 8]. Two workers exceeded a radiation exposure of 500 mSv, and four workers exceeded 250 mSv with mostly internal exposure [7, 8]. For the 3745 workers who participated in the emergency response in March 2011, the maximum and the average doses were 678.08 mSv and 31.13 mSv for external and internal radiation exposure, respectively [9].

Haematological methods are useful for evaluating the effects of ionizing radiation (IR) exposure on radiosensitive systems in the human body. In fact, substantial dose effects were observed among Mayak Production Association (PA) nuclear workers and Chernobyl clean-up worker cohorts using multicolour fluorescence *in situ* hybridization (mFISH), which is used for chronic or retrospective dose estimation in subjects to detect stable translocations [10–12]. As a haematological method, differential blood cell counts are also used to evaluate health conditions after IR exposure [10, 13]. Based on the aforementioned scientific evidence, 2164 Tokyo Electric Power Company (TEPCO) workers in this study participated in obligatory medical examinations before and after the FDNPP accident. Here, we analysed the haematological data to evaluate the effects of internal and external exposure and cofounding factors during the emergency period of the FDNPP accident.

## MATERIALS AND METHODS

### Study design and participants

All 2164 participants agreed to share their biological data for third-party evaluation and submitted written informed consent.

According to the ‘Ordinance on Prevention of Ionizing Radiation Hazards’ enacted by the Ministry of Health, Labour and Welfare to protect workers from health hazards due to radiation, all 2164 male emergency workers in this study underwent periodic (in the spring or autumn) medical examinations from 30 March 2006 through 15 January 2013. During this time, data from a total of 26 404 periodic medical examinations of 2164 TEPCO workers included 10 490 blood cell count data points from 1742 workers and 19 116 blood cell tests from 2164 workers (Table 1) [14]. Note that these reference values may differ across research institutions, and the reference range of each inspection agency was not applied in TEPCO internal health management. In addition, the time of blood transfer may vary across research institutions. Thus, one would expect that these variations in blood components to be randomly distributed among TEPCO workers exposed to low to high doses of radiation. The names and addresses of the participants were unknown, and the data were indicated with anonymous numbers. In addition, we had limited access to information on health conditions at blood sampling, work hours, workdays, work locations and medical exposure of the participants during the emergency period.

**Table 1:** Parameters obtained by periodic medical examinations

Parameters	Unit	Physical standard	Physical standard in TEPCO
1. Calendar year of medical examination	Year		
2. Working period	Day, Month		
3. Identification number (ID)	Number		
4. Date of periodic medical examination	Day, Month, Year		
5. Age at the time of medical examination	Year		
6. Cigarettes smoked per day	Number		
7. Quantity of alcohol consumed per day	Either a glass of sake (180 ml) or a bottle of beer (633 ml) set as one unit		
8. Frequency of alcohol consumption per month	Number		
9. White blood cell (WBC) counts	WBC /μL	4–10 ×10^3^/μL	3.2–7.7 ×10^3^/μL
10. Haemoglobin (Hb)	g/dL	14–18 g/dL for men	13.5–17.5 g/dL for men
11. Red blood cell (RBC) counts	RBC /μL	4.1–5.8 ×10^6^/μL	4.3–5.6 ×10^6^/μL
12. Haematocrit (Ht)	%	40–51%	41.1–53.9%
13. Lymphocyte (Lym)	% or /μL	18–58%	
14. Monocyte (Mono)	%	2–12%	
15. Neutrophil (Neu)	% or /μL	28–72%	
16. Stabs (Stab)	%	0–18%	
17. Segmented cells (Seg)	%	22–72%	
18. Eosinophil (Eos)	%	0–9%	
19. Basophil (Baso)	%	0–3%	
20. Atypical lymphocyte (A-lym)	%	0–2%	
21. Others			

^*^Note that these reference values may differ across research institutions. We refer to the ‘clinical method’ for the physiological standard of blood cell counts [14].

### Acquisition of dosimetric records

**Fig. 1:**
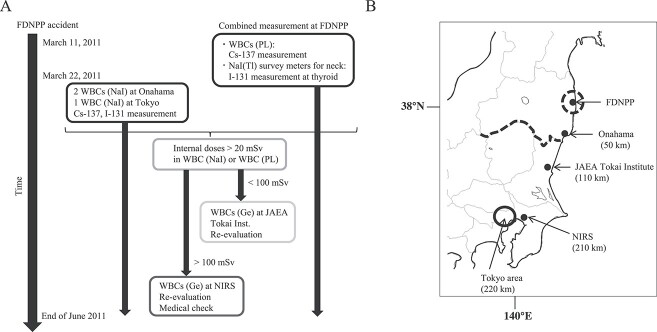
Internal exposure measurement procedure and locations during the emergency period. (A) Workflow of the internal exposure measurement procedure performed by TEPCO during the emergency period. PL: plastic scintillation detector; WBC: whole-body counter; NaI(Tl): thallium-doped sodium iodide; Ge: germanium semiconductor detector, JAEA: Japan Atomic Energy Agency; and NIRS: National Institute of Radiological Sciences. Please refer to the Methods section for precise information. (B) Surveyed locations for internal exposure during the emergency period. The Japan map focuses on the Tohoku (northeastern) area, where the FDNPP is located, and the Kanto region, where the capital, Tokyo, is located. The dotted line shows the border between the Tohoku area and the Kanto region. The numbers in parentheses indicate the distance from the FDNPP (km). The circle indicates the Tokyo area. The red circle indicates the area 20 km from the FDNPP. Faint lines indicate the borders between prefectures.

The tsunami caused a shortage of whole-body counters (WBCs) in damaged plants, and TEPCO obtained limited internal exposure data for emergency workers [15]. In addition, only 320 out of 5000 alarm pocket dosimeters (APDs) could be used for evaluating external exposure levels until April 1, 2011. A total of 640 APDs were supported by the Kashiwazaki-Kariwa Nuclear Power Station (Fig. 1), and 100 APDs were newly procured; and all workers started carrying APDs (1060 in total) after 1 April 2011. Therefore, the external dose data of the leader of each operational group was used as a representative data for all members [6], and some external dose data from March 2011 were estimated doses rather than actual values.

Beginning on 22 March 2011, TEPCO began operating two vehicle-mounted WBCs borrowed from the Japan Atomic Energy Agency (JAEA) at the Onahama Call Centre, 50 km away from the FDNPP and one vehicle-mounted WBC borrowed from JAEA in Tokyo, 220 km away from the FDNPP (Fig. 1). This geographical difference and the continuous increase in the number of workers at the FDNPP made it difficult for TEPCO to monitor all of the emergency workers and resulted in a delay in exposure monitoring [15]. Internal exposure data during the emergency period (until June 2011) were obtained using two types of WBCs, namely, thallium-doped sodium iodide (NaI(Tl), hereafter called ‘NaI’ for WBC for simplicity) stand-type scintillation detectors (FASTSCAN, Canberra Inc., USA) and chair-type plastic (PL) scintillation detectors (Fuji Electric Co., Ltd., Tokyo, Japan); the latter type of detector could not detect I-131, so compensatory I-131 measurement was conducted by focusing on the neck using a NaI(Tl) scintillation survey detector until the beginning of May 2011. The measured values by NaI(Tl) survey meters were multiplied by the thyroid deposition coefficient to evaluate thyroid I-131 intake. First, the WBC (PL) was calibrated using a solid radiation source of Cs-137 and anthropomorphic phantoms. Second, the calibration constant obtained from the phantoms was used for converting WBC (PL) net counts to Cs-137 equivalent whole-body volume. Third, the intake was calculated using the total body retention rate at the elapsed days from the assumed ingestion date (the work start date and the intermediate work date were set as the assumed ingestion date in March/April 2011 and in May or later in 2011, respectively). Fourth, the committed effective dose was calculated by multiplying the effective dose coefficient of Cs-137. WBC (PL) could not discriminate the WBC of Cs-134 and Cs-137 separately, and net signals were considered to be those from Cs-137. This may have caused overestimations for the committed effective dose from Cs-134 and Cs-137, but a conservative approach was chosen [16]. Finally, the committed effective dose from I-131 by the NaI(Tl) survey meter was added to the committed effective dose from Cs-134 and Cs-137. These sequential procedures to evaluate internal exposure were performed until the end of June 2011 (Fig. 1A). Exceptionally, the NaI(Tl) survey meter was used until early May 2011 because the count of I-131 for emergency workers was not detectable after early May 2011 due to its rapid half-life. WBCs with NaI scintillators were calibrated using a transfer phantom (Canberra Inc., USA) with the whole-body geometry source for assuming a uniform distribution of the nuclides throughout the entire body. Therefore, WBC (NaI) can identify and quantify radionuclides including I-131 and Cs-134 as the total body content rather than the thyroid content for I-131 in emergency workers. Effective doses for inhalation of radionuclides were calculated using effective dose coefficients (2.0 × 10^−5^ mSv/Bq for I-131, 9.1 × 10^−6^ mSv/Bq for Cs-134 and 9.7 × 10^−6^ mSv/Bq for Cs-137) [17]. Although the quantity of the effective dose is not directly measurable, we used values obtained by devices equipped during the emergency period for evaluating effective doses for individual workers. Due to confusion during the emergency period, tertiary evaluation for emergency workers was needed to obtain more accurate committed effective doses for some emergency workers [18].

Those exposed to more than 20 mSv as determined by WBC (PL) and WBC (NaI) measurements required additional monitoring after a 2-week interval to remove external contamination at the JAEA Tokai Institute 110 km away from the FDNPP using germanium (Ge) semiconductor detectors for more precise radionuclide identification and radioactivity measurement (Fig. 1). In addition, those exposed to more than 100 mSv required both medical examination by physicians and an internal exposure evaluation, and they were therefore dispatched to the National Institute of Radiation Sciences (NIRS) 210 km away from the FDNPP (Fig. 1). The internal exposure of at least 33 workers, including TEPCO workers, fell within the indicated criteria, and these workers were re-evaluated by WBC (Ge) at either the NIRS or JAEA Tokai Institute [15]. When TEPCO industrial physicians further examined 229 workers who had taken either more than 20 iodine tablets or tablets for more than 14 days continuously, they diagnosed four of these workers (1.8%) with hypothyroidism [19]. However, this hypothyroidism frequency was within the range of spontaneous hypothyroidism for adult men (1–8%) [20].

### Internal exposure effects

**Fig. 2:**
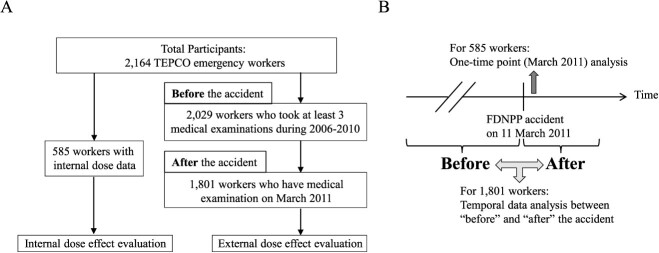
Flow chart for evaluation of internal and external dose effects of the medical examination data of emergency workers. **(**A) Workflow of internal dose effect evaluation of 585 TEPCO emergency workers who had internal dose data and workflow of external dose effect evaluation of 1801 TEPCO emergency workers who had at least three medical examinations during 2006–2010 (serving as data from before the accident) and a medical examination in March 2010 with external dose data (serving as data after the accident). (B) An analysis at one time point (in March 2011) for 585 workers was conducted to evaluate internal dose effects. A time-series data analysis for 1801 workers was conducted to evaluate external dose effects.

We used all available internal exposure dose records (mSv) of 585 workers who were exposed to relatively high doses as compared to background level (Max: 590 mSv, Min: 2.08 mSv, Mean: 19.7 mSv) (Fig. 2A). The estimated effective dose was the sum of the effective dose from external exposure and the effective dose from inhalation of radionuclides during the evaluation period. Radioiodine inhalation yielded most of the effective doses. The organ doses from radioiodine were calculated by integrating the retention in each organ according to ICRP biokinetic models [17]. To investigate the effects of thyroid doses, bone marrow (BM) doses, or the frequency of smoking or alcohol consumption on blood cells, we conducted log-linear model linear regression analyses.

### External exposure effects

#### Comparison of the measured values before and after the FDNPP accident

The external exposure data considered that the whole body had been evenly exposed. We first collected the data of 2029 workers who had at least three out of 10 medical examinations from 2006 through 2011 (Fig. 2A). Next, the data on the external exposure of 1801 workers were calculated as accumulated doses from March 2011 until the first medical examination (July 2011, Table 2). The subjects were all male and most of them were workers in their 30s to 50s. One third of the workers had external exposure doses of 0 mSv, and 20–30 workers had doses exceeding 100 mSv. Internal exposure was almost negligible. Periodic medical examinations were mandatory for workers registered in the FDNPP. The reason why 16.8% of workers had fewer than three medical examinations is that TEPCO workers from other branches who worked at nearby thermal power stations were immediately recruited to the FDNPP during the emergency period to support the emergency situation (according to personal communication with occupational health physicians working during the emergency period). We used a Wilcoxon signed-rank test to compare the measured value among the averages of at least three medical examinations and the medical examination in March 2011 (Fig. 2B, Table 3). The results were considered statistically significant at a *P* value of <0.05. There were few A-lym data; thus, we excluded this measure.

**Table 2:** Baseline characteristics

Number of consenting participants	2164							
		n	%	Mean ± S.D.		n	%	Mean ± S.D.
	Spring 2011	1801	83.2	-	Spring 2012	2148	99.3	-
Characteristic								
Sex	Male	1801	100	-	Male	2148	100	-
	Female	0	0	-	Female	0	0	-
Age (years)	<25	70	3.9	23.5 ± 1.1	<25	142	6.6	23.4 ± 1.4
	26–30	73	4.1	28.3 ± 1.4	26–30	163	7.6	28.0 ± 1.4
	31–35	131	7.3	33.5 ± 1.5	31–35	216	10.1	33.1 ± 1.4
	36–40	408	22.7	38.0 ± 1.4	36–40	397	18.5	38.2 ± 1.4
	41–45	456	25.3	43.0 ± 1.4	41–45	462	21.5	43.1 ± 1.4
	46–50	341	18.9	48.0 ± 1.4	46–50	371	17.3	48.0 ± 1.5
	51–55	270	15.0	52.7 ± 1.4	51–55	300	14.0	52.8 ± 1.3
	>55	52	2.9	56.9 ± 1.4	>55	97	4.5	57.1 ± 1.4
External dose (mSv)	0	599	33.3	-	0	106	4.9	-
	<10	554	30.8	3.9 ± 3.4	<10	1186	55.2	2.7 ± 3.1
	10–50	522	29.0	21.0 ± 10.1	10–50	644	30.0	21.9 ± 10.7
	50–100	104	5.8	68.7 ± 14.1	50–100	179	8.3	68.4 ± 13.7
	>100	22	1.2	119.1 ± 20.6	>100	33	1.5	118.0 ± 18.1
Internal dose^a^ (mSv)	0	1290	71.6	-	0	1556	72.4	-
	<10	218	12.1	5.5 ± 2.3	<10	257	11.9	5.5 ± 2.3
	10–50	269	14.9	20.0 ± 8.4	10–50	307	14.3	20.0 ± 8.6
	50–100	15	0.8	69.2 ± 14.3	50–100	19	0.9	67.2 ± 13.6
	>100	9	0.5	291.6 ± 182.8	>100	9	0.4	291.6 ± 182.8
Confounding factors								
Cigarettes smoked per day	0	998	55.4	-	0	1372	63.9	-
	1–10	266	14.8	6.3 ± 3.1	1–10	287	13.4	8.3 ± 2.6
	11–20	443	24.6	16.8 ± 3.1	11–20	452	21.0	17.9 ± 2.6
	21–30	81	4.5	24.0 ± 3.2	21–30	31	1.4	28.7 ± 2.2
	31–40	12	0.7	33.4 ± 2.9	31–40	6	0.3	39.2 ± 2.0
	41–50	1	0.06	-	41–50	0	0	-
Quantity of alcohol consumed per month	<1	125	6.9	-	<1	211	9.8	-
	1–20	952	52.9	8.4 ± 5.7	1–20	1242	57.8	8.0 ± 6.2
	21–40	394	21.9	29.2 ± 5.7	21–40	340	15.8	31.1 ± 5.9
	41–60	249	13.8	51.2 ± 6.1	41–60	254	11.8	55.5 ± 5.4
	>60	86	4.8	79.2 ± 13.3	>60	101	4.7	86.9 ± 26.4

^a^We used internal dose data until 2013 due to the difficulty of data collection during the emergency period (Fig. 1).

**Table 3:** Comparison of parameters (blood cell test data and confounding factors) before and after the FDNPP accident by a Wilcoxon signed-rank test

	Average of 2006–2010		Spring 2011		
Parameters	n	Mean ± S.D.	n	Mean ± S.D.	*P*-value
Cigarettes smoked per day	1801	6.4 ± 8.5	1797	5.7 ± 8.4	< 0.001
Frequency of alcohol consumption^a^	1801	21.6 ± 21.3	1798	19.8 ± 22.6	< 0.001
WBC^b^	1801	6011 ± 1394	1801	6036 ± 1593	0.266
Hb^c^	1801	15.3 ± 0.9	1801	15.2 ± 1.0	0.547
RBC^d^	1801	497 ± 31.8	1801	497 ± 35.3	0.714
Haematocrit %	1801	46.3 ± 2.4	1801	46.2 ± 2.8	0.058
Lymphocyte %	836	35.1 ± 6.8	1092	35.3 ± 7.8	0.117
Monocyte %	837	5.3 ± 1.3	1092	5.5 ± 1.6	< 0.001
Eosinophil %	837	2.8 ± 1.8	1092	3.0 ± 2.2	< 0.001
Basophil %	836	0.5 ± 0.4	1091	0.6 ± 0.6	< 0.001
Neutrophil %	834	56.4 ± 7.3	1068	55.6 ± 8.3	0.148

^a^Frequency of alcohol consumption: quantity of alcohol consumed per day × frequency of alcohol consumption per month

^b^White blood cells/μL

^c^Hb: gram of haemoglobin/dL

^d^10^4^ × red blood cells/μL

#### Correlation between accumulated external dose and variation in each blood cell count

Our data indicated a nonnormal distribution; thus, we computed a nonparametric bivariate correlation, namely, Spearman’s rank correlation coefficient, to examine the degree of association between the two variables and the direction of the relationship (Table 4). This test carries no assumptions on the data distribution and, hence, is appropriate for analysis when the variables are measured on an ordinal scale.

**Table 4:** Correlation coefficient between external exposure dose and each variable obtained by Spearman’s rank correlation coefficient

		Spearman	
Variable	n	Correlation coefficient	*P*-value
Age	1801	−0.043	0.071
DrinkDif	1798	−0.059	0.012
SmokeDif	1797	0.038	0.106
WBCDif	1801	0.034	0.154
HbDif	1801	0.122	< 0.001
RBCDif	1801	0.049	0.037
HtDif	1801	−0.046	0.052
LymDif	736	−0.031	0.401
MonoDif	736	0.052	0.155
EosDif	736	0.069	0.065
BasoDif	736	0.032	0.439

Dif: Difference variation (after the accident – before the accident)

#### Dependence and relation between significantly altered factors and explanatory variables based on the results of bivariate correlations

To find an independent variable group with strong dependence and relation in time-series analysis (Fig. 2B), we conducted criterion-based procedures with the Akaike information criterion (AIC) for variable selection in the generalized linear model (GLM) [21]. Competing models with these variables are ranked according to their AIC value, and the model with the lowest AIC value is considered the best-fitting statistical model. Note that the AIC can relatively measure the goodness of fit of the model, and there is no established value above which a given model is rejected. We selected criteria for variables in which a significance level below 0.11% in Spearman’s rank correlation coefficient was selected (Table 4). Age, alcohol consumption frequency, smoking per day, haemoglobin (Hb), red blood cell (RBC), haematocrit (Ht) and eosinophils (Eos) met these criteria. First, we set the exposure dose as an objective variable, while age, alcohol consumption frequency, smoking per day and Hb were used as explanatory variables. Since RBC and Ht had strong correlations with Hb and behaved as intervening variables, they were excluded from this analysis [14]. The sample size of Eos was lower than that of the rest of the values and hence excluded (Table 5). Therefore, the formula was as follows:


$$\begin{array}{l} \mathrm{GLM}\ \left(\mathrm{formula}=\mathrm{EE}\ (\mathrm{exposure}\ \mathrm{effect}\right)\sim \mathrm{Age}2011\\+\mathrm{SmokingDif}+\mathrm{DrinkingDif}+\mathrm{HbDif}) \end{array}$$

**Table 5:** Estimation between external exposure dose and significantly altered variables in Table 3 using criterion-based procedures with the AIC for variable selection in the GLM

Variable	Parameter estimate	Standard error	t-value	Pr (> |t|)
Intercept	17.74	2.78	6.38	2.21e^−10^
Age2011	−0.12	0.064	−1.93	0.054
DrinkDif	−0.15	0.035	4.15	3.42e^−5^
HbDif	5.17	0.077	6.68	3.21e^−11^

In the formula, Hb was set as the objective variable, while exposure dose, age, frequency of alcohol consumption and smoking per day were set as explanatory variables as follows:


$$\begin{array}{l} \mathrm{GLM}\ (\mathrm{formula}=\mathrm{HbDif}\sim \mathrm{EE}+\mathrm{Age}2011\\+\mathrm{SmokingDif} +\mathrm{DrinkingDif})\end{array}$$

The Hb change ratio (%) was analysed by grouping the values into four external dose ranges (> 100 mSv, 50–100 mSv, 10–50 mSv, < 10 mSv) in emergency workers and performing linear regression analysis. We conducted five different analyses as listed in Table 6.

**Table 6:** Five different linear regression analyses for the Hb change ratio (%) by grouping the values into four external dose ranges (> 100 mSv, 50–100 mSv, 10–50 mSv, < 10 mSv) in emergency workers

1	100 × [Hb average (2011 and 2012, spring and autumn) – Hb average (2006–2010, spring and autumn)]/Hb average (2006–2010, spring and autumn)
2	100 × [Hb average (2011, spring and autumn) – Hb average (2006–2010, spring and autumn)]/Hb average (2006–2010, spring and autumn)
3	100 × [Hb average (2012, spring and autumn) – Hb average (2006–2010, spring and autumn)]/Hb average (2006–2010, spring and autumn)
4	100 × [Hb average (2011 and 2012, spring) – Hb average (2006–2010, spring)] / Hb average (2006–2010, spring)
5	100 × [Hb average (2011 and 2012, autumn) – Hb average (2006–2010, autumn)] / Hb average (2006–2010, autumn).

### Statistical analysis software

Statistical analyses were performed using SAS and JMP software (SAS Institute Inc., Cary, NC, USA) and the computing environment R.

### Ethics

Data acquisition for this study was performed after approval was granted by the Ethics Review Committee of the University of Occupational and Environmental Health, Japan (H24-118).

## RESULTS

### Evaluation of the internal dose effect

Since an evaluation of health effects on internally exposed emergency workers was indispensable, we first focused on the 22 TEPCO workers who had been internally exposed to more than 20 mSv and were dispatched to either the JAEA or NIRS to receive re-evaluation with high-resolution WBC (Ge) (Fig. 1, Table 7). When we considered the effective dose coefficients for Cs-134 and Cs-137 [17], the Cs-134/Cs-137 ratios (≈ 1.0) among emergency workers were compatible to the previously reported ratios (≈ 1.0) during March 2011 (Table 7) [22]. Given the period of at least 2 weeks between the first and second checks by WBC (Ge), we estimated that these emergency workers had inhaled short half-life I-131 rather than Cs-134 and Cs-137 soon after the accident. Indeed, when we analysed these data from six workers with external and internal exposure data from several months later, rapid decreases in internal exposure were observed (Table 8). Therefore, we decided to focus on the effect of volatile I-131 on internal exposure as a major inhalation contamination substrate for emergency workers; we did not consider committed effective doses of I-131 because of its short effective half-life (8 days) during the emergency period in March 2011 [1].

**Table 7:** Internal doses of workers who received re-evaluation by WBC(Ge)

				Internal dose (mSv)			
Number of workers^a^	Year/month^b^	External dose (mSv)	Measuring instrument	I-131	Cs-134	Cs-137	Total
1	2011/03	99.37	WBC(Ge)^c^	543.35	1.72	1.20	546.27
2	2011/03	80.37	WBC(Ge)	586.67	4.59	3.26	594.52
3	2011/03	29.75	WBC(Ge)	259.11	0.32	0.23	259.66
4	2011/03	74.30	WBC(Ge)	39.95	0.56	0.41	40.92
5	2011/03	43.87	WBC(Ge)	82.80	1.05	0.73	84.58
6	2011/03	36.44	WBC(Ge)	135.96	0.77	0.53	137.26
7	2011/03	96.44	WBC(Ge)	57.84	1.23	0.86	59.93
8	2011/03	56.11	WBC(Ge)	49.60	0.51	0.37	50.48
9	2011/03	114.96	WBC(Ge)	22.52	0.4	0.29	23.21
10	2011/03	69.17	WBC(Ge)	18.27	0.8	0.56	19.63
11	2011/03	57.45	WBC(Ge)	47.73	0.35	0.25	48.33
12	2011/03	104.46	WBC(Ge)	240.38	0.85	0.58	241.81
13	2011/03	43.66	WBC(Ge)	26.02	0.32	0.23	26.57
14	2011/03	53.52	WBC(Ge)	82.36	1.02	0.68	84.06
15	2011/03	62.72	WBC(Ge)	56.98	0.2	0.15	57.33
16	2011/03	38.13	WBC(Ge)	56.67	0.6	0.40	57.67
17	2011/03	71.08	WBC(Ge)	96.35	0.38	0.28	97.01
18	2011/03	31.34	WBC(Ge)	60.54	1.68	1.20	63.42
19	2011/03	68.33	WBC(Ge)	17.11	0.29	0.22	17.62
20	2011/03	25.67	WBC(Ge)	432.75	0.17	0.13	433.05
21	2011/03	26.41	WBC(Ge)	165.73	0.19	0.13	166.05
22	2011/03	13.82	WBC(Ge)	119.06	0.31	0.22	119.59

^a^Among those who exposed to more than 20 mSv, 22 TEPCO emergency workers listed here dispatched to either JAEA or NIRS to receive re-evaluation with WBC(Ge) (Methods).

^b^The date was supposed to be at least 2 weeks later after the 1^st^ check (Methods).

^c^Whole body counter with germanium semiconductor detector.

**Table 8:** Comparison of internal and external exposure doses for 6 workers between March 2011 and the month of re-evaluation in 2011

Number of workers^a^	Year/month	External dose (mSv)	Internal dose (mSv)	Re-evaluation year/month^b^	External dose (mSv)	Internal dose (mSv)
1	2011/03	45.03	36.4	2011/05	12.98	4.56
2	2011/03	16.12	77.7	2011/06	0.43	2.96
3	2011/03	32.39	19.4	2011/08	13.96	2.66
4	2011/03	106.03	39.3	2011/05	10.22	2.47
5	2011/03	5.42	18.1	2011/07	3.94	4.32
6	2011/03	32.00	45.6	2011/06	1.18	3.72

^a^Among those who were exposed to more than 20 mSv, 6 TEPCO emergency workers listed here were dispatched to either JAEA or NIRS to receive re-evaluation with WBC(Ge) (Methods).

Note that we have no information regarding whether these workers worked again at the FDNPP after 2011/3, and we cannot exclude the possibility of re-exposure after the accident.

^b^We have no information on the reason why only these 6 workers have data in both March 2011 and in the month of re-evaluation of 2011.

**Fig. 3:**
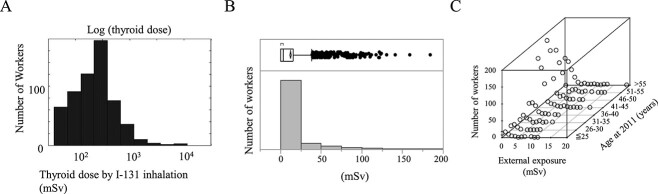
Evaluation of internal dose effects for those who were internally exposed and evaluation of the external exposure dose in March 2011. **(**A) Histogram of thyroid dose due to inhalation of I-131. Thyroid doses are shown logarithmically from 10^2^ mGy to 10^4^ mGy. (B) The external exposure of 2104 emergency workers was 12.3 ± 0.45 (SE) mSv and 12.3 ± 20.6 (SD) mSv. The minimum dose was 0 in 688 workers (31.8%). The maximum dose was 184.8 mSv, with a median of 3.4 mSv. (C) There was no correlation between age and exposure dose. External exposure (EE) = 12.13 + 0.003* Age; R = 0.0013 (−0.0415, 0.0440). The correlation coefficient was 0.01; 95% CI: −0.03–0.05.

We collected information for 585 out of 2164 workers from whom internal exposure data were obtained in March 2011 and calculated either thyroid dose or BM dose by I-131 inhalation. In the histogram of thyroid doses, we observed high-dose exposed workers with as much as 10 Sv of exposure in the thyroid (Fig. 3A). In contrast, in the histogram of BM doses, the dose was estimated to be below 0.1 mSv for most workers (Fig. S1). Although the equivalent dose of BM was four orders of magnitude lower than that of the thyroid, BM is essential for understanding radiation physiology, and we used linear regression analysis to examine the correlations between blood cell counts and the dose of radiosensitive BM where blood cells are formed [23]. Compared to the doses of external exposures, the doses of internal exposures were much lower, and we could not observe significant haematological changes with internal exposures except for significant positive correlations between smoking effects and WBCell (data not shown) [24]. Interestingly, BM dose was positively correlated with Hb when we evaluated the effect of internal plus external exposure (data not shown). To confirm this result, we corrected Hb values with smoking frequency, and again, Hb showed a significant positive correlation with total exposure doses (Fig. S2A), although Hb values were within the range of normal values for men of 14 to 18 g/dL. Conversely, when corrected with drinking frequency, Hb exhibited a significant negative correlation with total exposure (Fig. S2B). These results suggest that Hb was mainly correlated with the external exposure dose and that the correlation was susceptible to confounding factors, such as the frequency of alcohol consumption, among emergency workers.

### Comparison between before and after the FDNPP accident

Next, we selected 1801 TEPCO workers based on the criteria for evaluating external exposure effects (Fig. 2, Table 2). Their average age was 42.6 ± 7.9 years, and their ages were not correlated with the exposure dose (Fig. 3). The accumulated external doses of subjects were 0-184.8 mSv, and most workers were exposed to less than 25 mSv externally (Fig. 3). Significant decreases were observed in cigarettes smoked per day and the frequency of alcohol consumption (*P* < 0.001), while significant increases were observed in blood factors within the normal ranges, such as Mono (*P* < 0.001), Eos (*P* < 0.001) and Baso (*P* < 0.001) (Table 3). To confirm whether the effects of Hb and the frequency of alcohol consumption were present only during the early emergency period, we analysed the data collected before and in the autumn of 2011. Both cigarettes smoked per day and the frequency of alcohol consumption returned to the levels of before the accident. Surprisingly, the relationship between increased Hb and external exposure dose was sustained or even slightly increased towards the autumn of 2011 (data not shown).

Then, we examined the degree and direction of the association between the accumulated external exposure and each variable obtained by medical examinations among 1801 workers (Fig. 2, Table 4). While positive associations between the accumulated external dose and cigarettes smoked per day (*P* = 0.106), Hb (*P* < 0.001), RBC (*P* = 0.037) and Eos (*P* = 0.065) were recognized, a negative association between the accumulated external dose and age (*P* = 0.071), frequency of alcohol consumption (*P* = 0.012) and Ht (*P* = 0.052) was also observed. We next examined the effects of external exposure multilaterally. The variables Eos, RBC and Ht were eliminated by this analysis, and smoking status was excluded during the stepwise variable selection (AIC = 16 102). Hb had a strong positive relationship with the accumulated external dose (*P* = 3.21e^−11^; Table 5). In contrast, alcohol consumption frequency had a negative relationship with the accumulated external dose (*P* = 3.42e^−5^; Table 5). Since Hb variation exhibited the strongest correlation with external dose, we next focused on Hb variation as an objective variable (AIC = 3490). Although we recognized a positive relation between the variation in Hb and the variation in daily smoking (*P* = 4.51e^−5^; Table S1), the exposure dose effect showed a strong relationship with the variation in Hb variation (*P* = 1.86e^−10^; Table S1).

Finally, emergency workers were grouped into four external dose range categories: > 100 mSv, 50–100 mSv, 10–50 mSv and < 10 mSv and the Hb change ratio was assessed to maximize the usefulness of the pre- and post-FDNPP accident data. We found a positive relationship between the exposure dose and the Hb change ratio, particularly in the >100 mSv group followed by the 50-100 mSv group (Fig. S3). This trend was consistently observed regardless of season (spring or autumn) and single-year comparison (data not shown). In summary, these data suggest that the exposure doses showed certainly showed a positive correlation with Hb levels.

## DISCUSSION

This study used blood samples from 2614 TEPCO employees at the time of the FDNPP accident and compared blood sample data before and after the accident. Our study provides evidence that radiation exposure dose is correlated with blood cell counts and describes how confounding factors affected blood sample changes during the emergency period. In particular, we found an unexpected positive correlation between the exposure dose and Hb levels, albeit within the normal range.

Although 138 workers were exposed to more than 100 mSv of radiation before July 2011, most of the emergency workers in this study were externally exposed to less than 25 mSv and internally exposed to less than 0.1 mSv for BM doses (Figs 3 and S1). These exposure doses are much lower than the recommended value of 0.4 Gy per year provided by the ICRP to prevent haematopoietic dysfunction [25], and the possibility of haematopoietic dysfunction is quite low. Indeed, a Wilcoxon signed-rank test showed no alteration in the WBCell value and slight but significant changes in Mono, Eos and Baso within the range of normal values after the accident (Table 3). These blood cells have a role in the immune system, allergic responses and inflammation [26]; thus, we could detect a slight stimulation exerted by complex factors using a large data set.

We found that the frequency of alcohol consumption and cigarettes smoked per day decreased significantly after the accident (Table 3). Since these transiently decreased frequencies returned to the levels before the accident by the autumn of 2011 (data not shown), we speculate that emergency workers had fewer opportunities to purchase alcohol and tobacco or were unable to relax due to the tense situation during the emergency period [6, 27]. Notably, the frequency of alcohol consumption exhibited a more significant reduction than the frequency of smoking (Tables 3 and 4). During the emergency period, there were designated areas for smokers in the FDNPP during temporary breaks. On the other hand, alcohol consumption after daily emergency work can be disturbed by uncomfortable sleeping, eating, resting and sanitation conditions in a tense situation.

Internal thyroid exposure to I-131 was relatively high for some workers, but internal BM exposure was not. It is widely accepted that excessive alcohol consumption induces anaemia [28]. Indeed, the frequency of alcohol consumption was negatively correlated with Hb (Fig. S2B). In contrast, the correlation of cigarettes per day with increased WBCell and Hb has also been well known for decades [24, 29], confirming the power of the linear regression analysis in our study (Fig. S2). Unexpectedly, a positive correlation between BM doses and Hb levels was observed (Fig. S2). Similarly, Hb levels were positively correlated with external exposure doses (Tables 4 and 5). An evaluation for assessing confounding factors that may affect increased Hb levels revealed that only smoking frequency tended to be relevant to increased Hb levels after the FDNPP accident (Table S1).

Hb is the oxygen-transport metalloprotein in RBCs and is composed of haem and globin. While haem is synthesized in the mitochondria and cytosol of immature RBCs, globin is synthesized by ribosomes in the cytosol, which is enhanced in the BM [30]. However, increased Hb stimulated by a low dose of BM exposure seems unlikely because the BM doses in the majority of emergency workers were below 0.1 mSv (Fig. S1; [17]), although BM is one of the most radiosensitive organs in the body. In contrast, a high dose of I-131 exposure in emergency workers was clear in our study (Fig. 3A). Endocrine hormones, including thyroid hormone, have important effects on erythropoiesis in humans [31]. Since thyroid hormone can stimulate RBCs, Ht and Hb, no study has reported only increased Hb levels among RBCs, Ht and Hb after high-dose I-131 exposure for therapeutic purposes.

There are several possible reasons why the increased Hb levels were correlated with the external exposure doses in emergency workers associated with the tense situation during the emergency period. First, long-term unbalanced dietary intake may affect Hb levels. Only ready-made foods in retort pouches were available for emergency workers until boxed lunches with fresh food became available in September 2011 (Fig. S4). However, all emergency workers ingested similar foods, and it seems unlikely that long-term unbalanced dietary patterns altered haematological conditions associated with external exposure doses. Second, emergency workers were exposed to multiple stressors including additional explosions after the accident, which can affect Hb levels. Some workers had to work far from their evacuated families and/or lost their relatives because of the tsunami [6]. Furthermore, discrimination and slander increased the stress of TEPCO workers because the FDNPP belongs to the TEPCO [32]. These situations created an extremely adverse work environment from a psychological perspective. Several studies propose that acute mental stress is correlated with haemoconcentration characterized by the acute loss of plasma volume of the intravascular space, where Hb and Ht are concentrated [33], while our study obtained inverse relations between Hb and Ht levels and external exposure doses in emergency workers (Table 4). Interestingly, total Hb during the cognitive task in near-infrared spectroscopy (NIRS) was positively correlated with the scores in attention and concentration on the Wechsler Memory Scale-Revised in subjects with post-traumatic stress disorder (PTSD) among victims of the Tokyo subway sarin attack [34]. It should be noted that NIRS has technical limitations, including limited spatial resolution and interference from a variety of anatomical factors, and thus, whether PTSD can affect peripheral blood Hb levels in an external dose-dependent manner warrants further investigation. Third, a possible statistical problem is that outliers were larger for higher doses, and these large outliers caused the slope of Hb according to the exposure dose (Fig. S2). Fourth, a physical reason may underlie the increased Hb levels associated with the external exposure dose. The number of skilled and professional workers in the FDNPP emergency period was limited, and consequently, these workers tended to work long hours with respirator masks [6, 15]. Some might debate the safety benefit of respirator masks during the emergency period given the trade-off between the prevention of contaminant inhalation and the reduction in performance during prolonged wear [35]. Moreover, haemolysis and haemoglobinemia characterized by high haemoglobin counts are primary ATP-release mechanisms in human erythrocytes during exercise performed in hypoxia [36, 37]. Therefore, we assume that those exposed to higher doses installed their mask tightly for a longer time period than those exposed to lower doses during the emergency period. Along with this speculation, prolonged work with a respirator mask during the emergency period may lead to a higher Hb density per unit caused by dehydration. Dehydration preceding exercise in hot conditions magnifies thirst-driven drinking during exercise in the heat and results in increased Hb but not Ht levels in healthy men [38]. However, both Hb and Ht depend on plasma volume, and dehydration should cause higher Hb and Ht levels [14]. It is currently unclear why only Hb increased under exercise-related heat stress conditions (Table 4; [38]). Finally, the possibility of relative polycythaemia induced by stress in increment Hb levels can be considered because RBC and Hb levels showed a positively correlated trend with external exposure (Table 4; [39]). Notably, the aforementioned reasons and other unconsidered reasons may influence each other. For example, intermittent sleep disturbances in uncomfortable accommodations during the emergency period could affect haematological conditions. The long-lasting correlations between increment Hb and the external exposure dose until the autumn of 2011 were unexpected and interesting. At present, there are no reports to support this finding, and further study is needed to understand these relationships under unusual tense situations.

The Mayak PA workers and residents downstream of the Techa River were internally and externally exposed protractedly at low-dose-rate IR from 1949 until 1956 [40]. More than 90% of the total BM dose was due to internal beta-particle emitters in the Techa River case [41, 42]. The estimated BM dose by mainly internal exposure was at most 2 Gy with a mean of 0.30 Gy, whereas the BM dose by internal and external exposure in the FDNPP accident case ranged from 0.01 mGy to 1 mGy (Fig. S1).

A significant increase in Hb levels was evident among smokers compared to non-smokers in both the Adult Health Study and the Atomic Bomb Survivor Study [43]. Consistently, Hb levels associated with exposure increased with smoking (Fig. S2, Table S1). Strikingly, our study revealed that Hb levels also increased with BM dose (Fig. S2). Moreover, a significant increase in Hb levels associated with external exposure was observed, with a downward trend in smoking frequency (Table 3). These findings contradict the finding of an age-dependent decrease in Hb in atomic bomb survivors who were exposed to more than 1 Gy [43], although a longitudinal functional decline in haematopoietic stem cells may result. This discrepancy can be attributed to the difference between the effect of acute high-dose exposure by atomic bomb after a period exceeding 40 years and the effect of protracted low-dose exposure during the emergency period of the FDNPP accident.

Our study has several limitations. First, the statistical analysis in this study was adjusted only for measured variables, and unmeasured factors could be confounders. Although numerous acute and chronic diseases differentially affect peripheral blood cell composition, we have no information on the health condition of the workers at blood sampling. Second, different research institutions and blood transfer times for blood sampling would affect the haematological data of TEPCO workers. Third, we have no information on which emergency workers took iodine tablets or on those who left and returned to work at the FDNPP again during the emergency period. Forth, emergency help from TEPCO workers from nearby thermal power stations may have caused sampling bias. Finally, some doses of exposure among emergency workers were not exact but rather were estimated because of inappropriate monitoring due to a shortage of personal dosimeters and delayed internal exposure monitoring (Fig. 1; [15]). Therefore, our estimates may have led to an over- or underestimation of the risks.

Importantly, we had data on quantitative characteristics over the period (before and after the accident) concerning confounding factors, such as cigarettes smoked per day, which may have affected individual health conditions. Therefore, we could minimize the effects of these non-radiation confounders to evaluate health conditions during the emergency period. The availability of initial data by routine medical examinations several years prior to the FDNPP accident reinforced the control reference data before the FDNPP accident. This strength is essential for assessing the data after the accident. Moreover, this study describes unexpected health effects on FDNPP emergency workers by analysing large sample data consisting of internal and external doses combined with haematological data.

This study is the first unbiased health evaluation of the internal and external exposure of emergency workers following a nuclear disaster at the highest INES level of 7. This study may thus serve as a reference for evaluating the health conditions of occupationally exposed personnel with acute exposure caused by accidents.

## Supplementary Material

Supplemental_Figures_rrac089

## Data Availability

The data that support the findings of this study are available from the corresponding author upon reasonable request.

## PRESENTATION AT A CONFERENCE

Part of this study was presented at the 59th Annual Meeting of the Japanese Radiation Research Society and the 23rd Meeting of the Conference on Radiation & Health in 2016.
