# Supplementary material for: Relationship between haematological data and radiation doses of TEPCO workers before and after the FDNNP accident
Source: J Radiat Res. 2023 Jan 4;64(2):261–72. doi: 10.1093/jrr/rrac089 (PMC10599404; doi:10.1093/jrr/rrac089)
Supplement: Supplemental_Figures_rrac089 [file supplemental_figures_rrac089.pptx]

## Slide 1
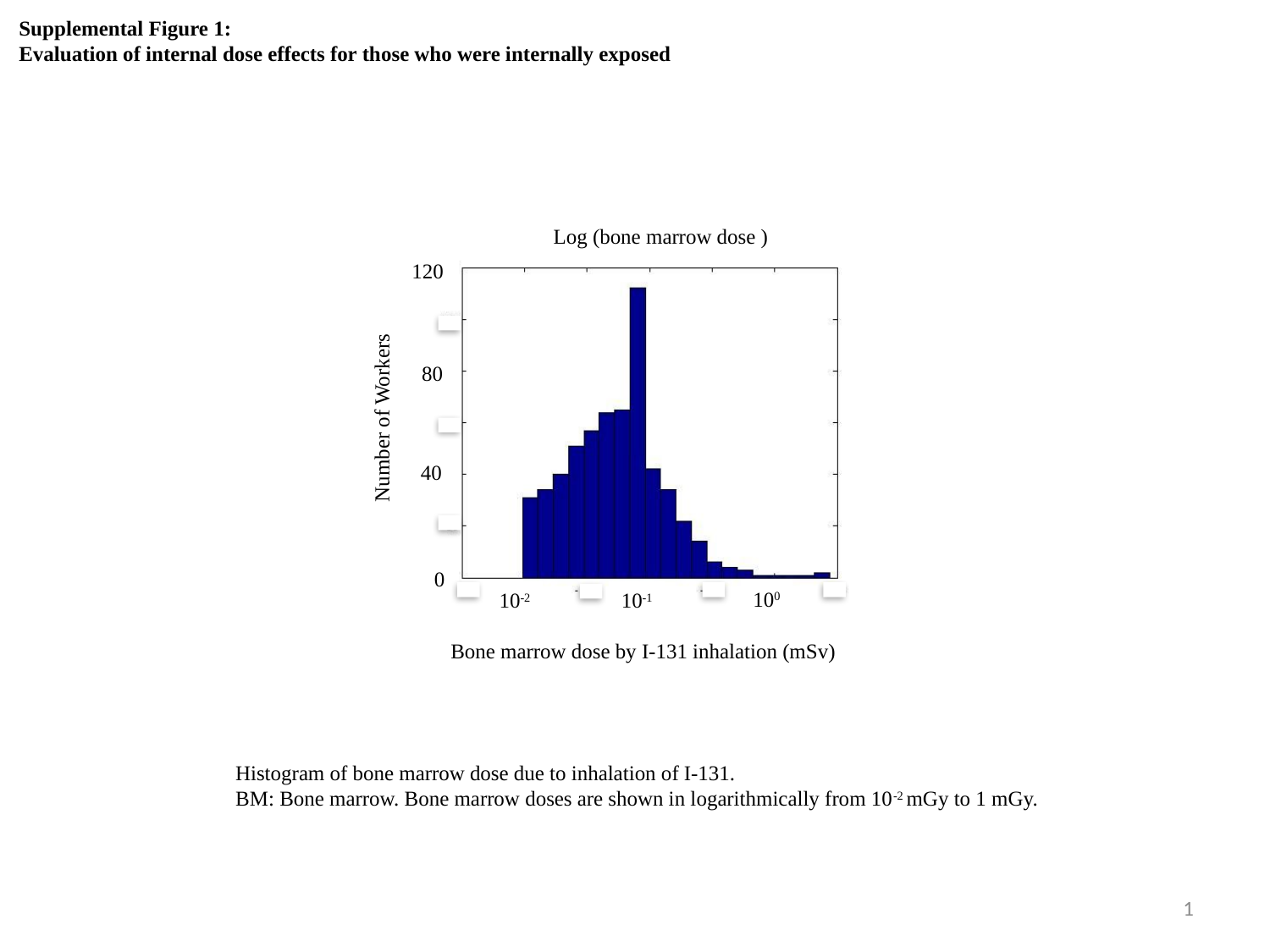

Supplemental Figure 1:
Evaluation of internal dose effects for those who were internally exposed
Log (bone marrow dose )
120
80
40
Number of Workers
0
100
10-2
10-1
Bone marrow dose by I-131 inhalation (mSv)
Histogram of bone marrow dose due to inhalation of I-131.
BM: Bone marrow. Bone marrow doses are shown in logarithmically from 10-2 mGy to 1 mGy.
1

## Slide 2
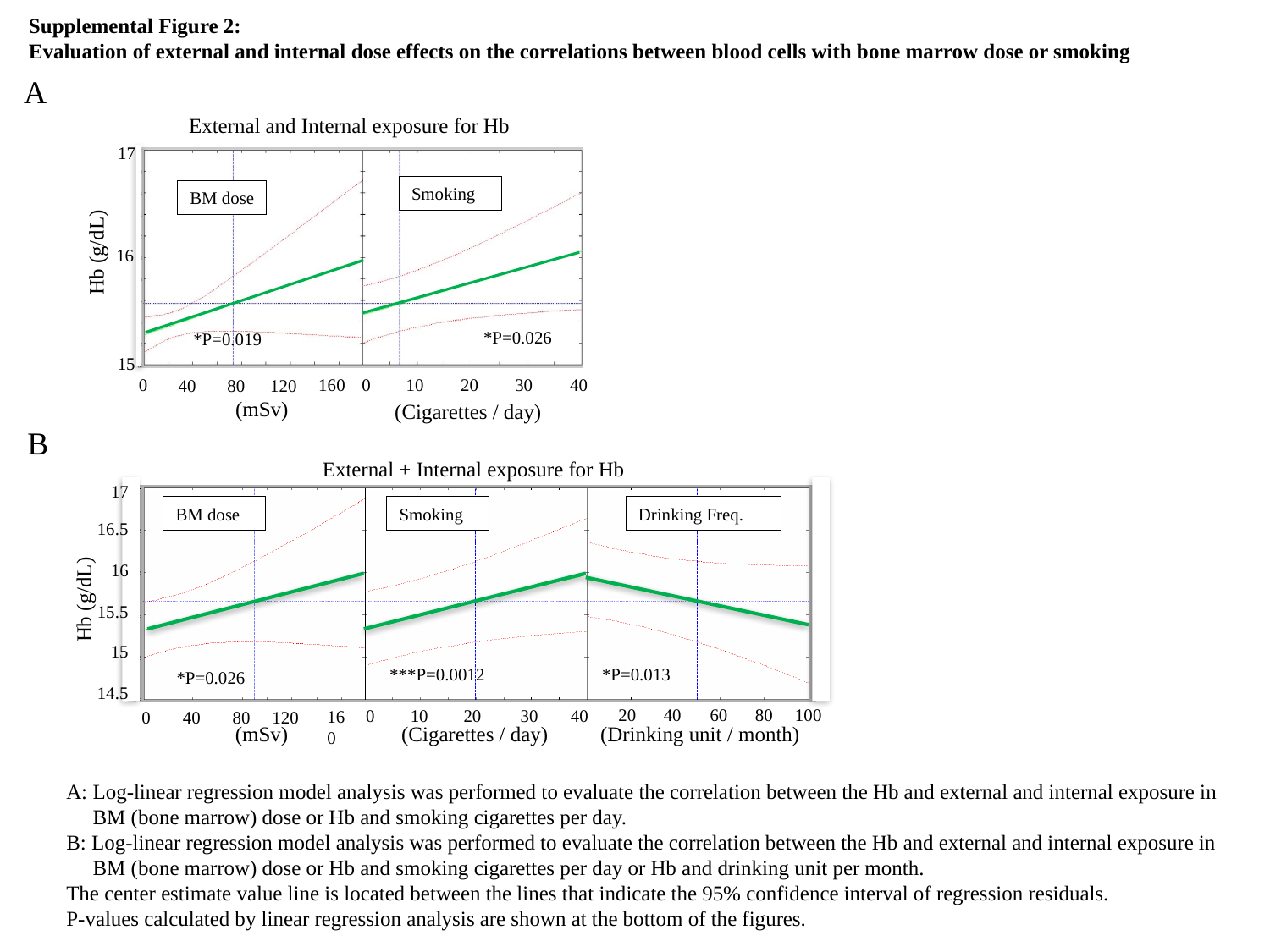

Supplemental Figure 2:
Evaluation of external and internal dose effects on the correlations between blood cells with bone marrow dose or smoking
A
External and Internal exposure for Hb
17
Smoking
BM dose
Hb (g/dL)
16
*P=0.026
*P=0.019
15
0
160
0
10
20
30
40
40
120
80
(mSv)
(Cigarettes / day)
B
External + Internal exposure for Hb
17
BM dose
Smoking
Drinking Freq.
16.5
16
Hb (g/dL)
15.5
15
***P=0.0012
*P=0.013
*P=0.026
14.5
20
40
60
80
100
0
10
20
30
40
0
40
80
120
160
(mSv)
(Cigarettes / day)
(Drinking unit / month)
A: Log-linear regression model analysis was performed to evaluate the correlation between the Hb and external and internal exposure in
 BM (bone marrow) dose or Hb and smoking cigarettes per day.
B: Log-linear regression model analysis was performed to evaluate the correlation between the Hb and external and internal exposure in
 BM (bone marrow) dose or Hb and smoking cigarettes per day or Hb and drinking unit per month.
The center estimate value line is located between the lines that indicate the 95% confidence interval of regression residuals.
P-values calculated by linear regression analysis are shown at the bottom of the figures.

## Slide 3
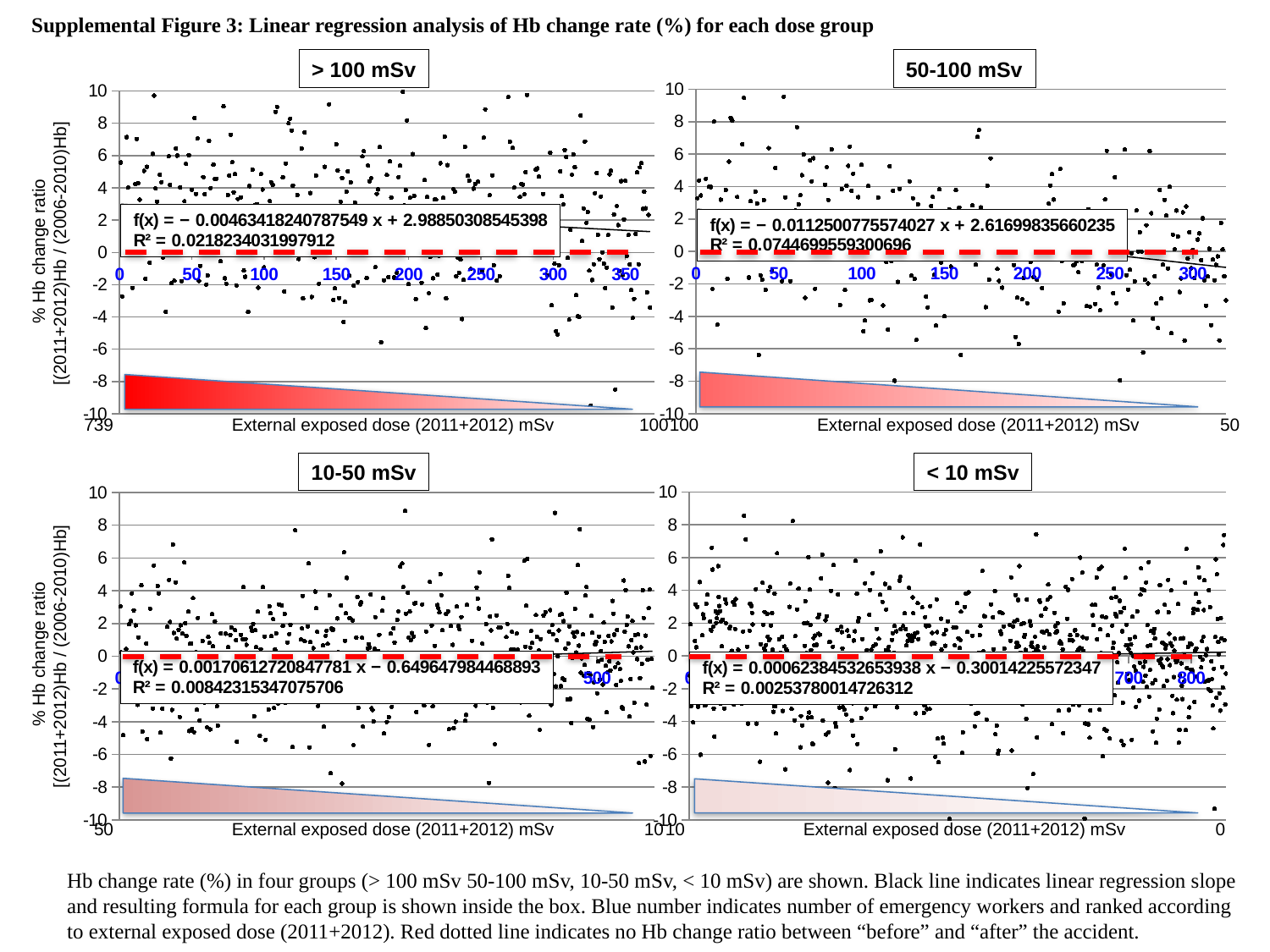

Supplemental Figure 3: Linear regression analysis of Hb change rate (%) for each dose group
> 100 mSv
50-100 mSv
### Chart
| Category | |
|---|---|
### Chart
| Category | |
|---|---| % Hb change ratio
 [(2011+2012)Hb / (2006-2010)Hb]
739　　　　　　 External exposed dose (2011+2012) mSv　　 100
100　　　　　 　External exposed dose (2011+2012) mSv　　 50
10-50 mSv
< 10 mSv
### Chart
| Category | |
|---|---|
### Chart
| Category | |
|---|---| % Hb change ratio
 [(2011+2012)Hb / (2006-2010)Hb]
50　　　　　 　External exposed dose (2011+2012) mSv　　 10
10　　　　　　 External exposed dose (2011+2012) mSv　　 0
Hb change rate (%) in four groups (> 100 mSv 50-100 mSv, 10-50 mSv, < 10 mSv) are shown. Black line indicates linear regression slope
and resulting formula for each group is shown inside the box. Blue number indicates number of emergency workers and ranked according
to external exposed dose (2011+2012). Red dotted line indicates no Hb change ratio between “before” and “after” the accident.

## Slide 4
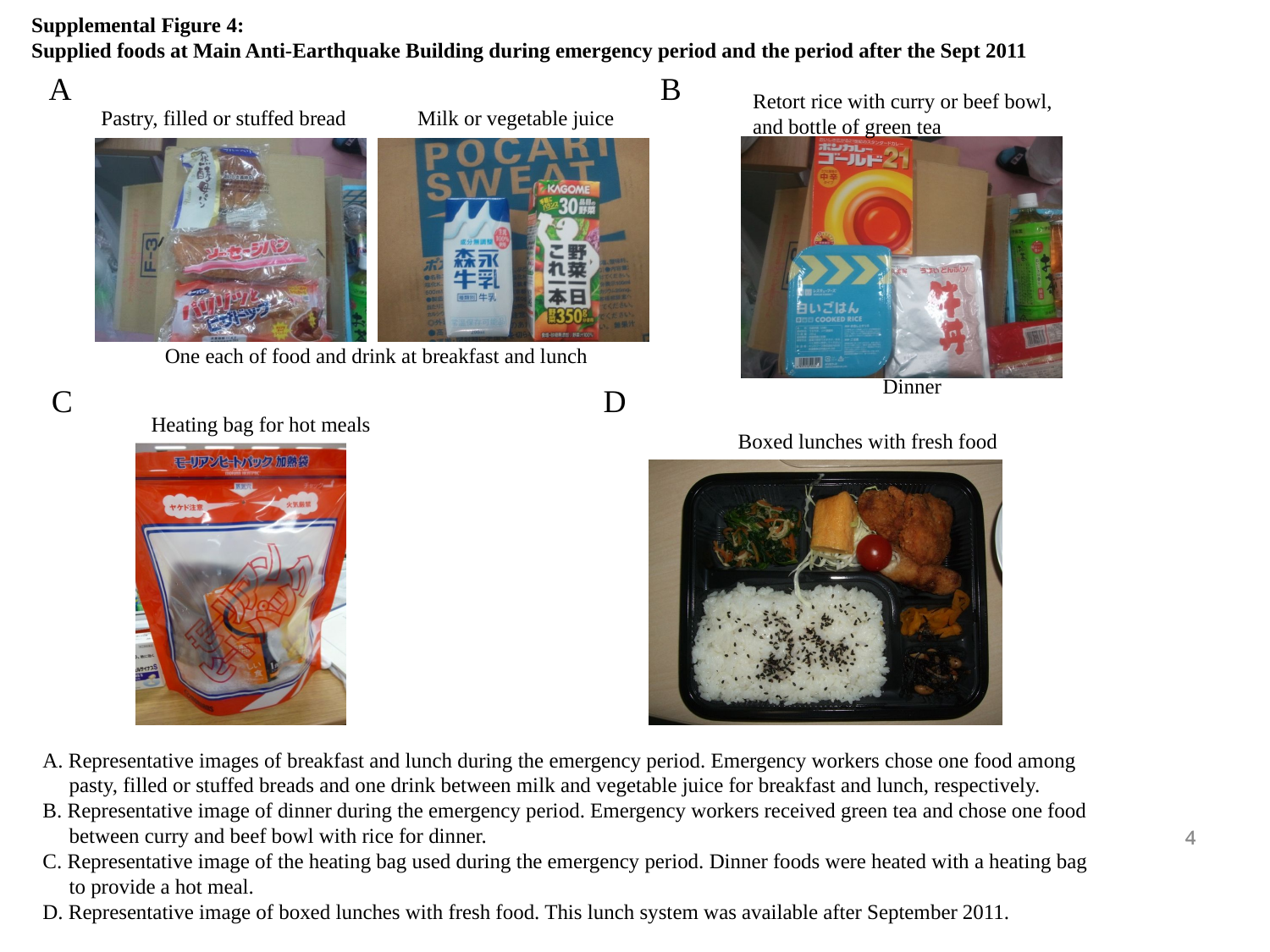

Supplemental Figure 4:
Supplied foods at Main Anti-Earthquake Building during emergency period and the period after the Sept 2011
A
B
Retort rice with curry or beef bowl,
and bottle of green tea
Milk or vegetable juice
Pastry, filled or stuffed bread
One each of food and drink at breakfast and lunch
Dinner
C
D
Heating bag for hot meals
Boxed lunches with fresh food
A. Representative images of breakfast and lunch during the emergency period. Emergency workers chose one food among
 pasty, filled or stuffed breads and one drink between milk and vegetable juice for breakfast and lunch, respectively.
B. Representative image of dinner during the emergency period. Emergency workers received green tea and chose one food
 between curry and beef bowl with rice for dinner.
C. Representative image of the heating bag used during the emergency period. Dinner foods were heated with a heating bag
 to provide a hot meal.
D. Representative image of boxed lunches with fresh food. This lunch system was available after September 2011.
4
4

## Slide 5
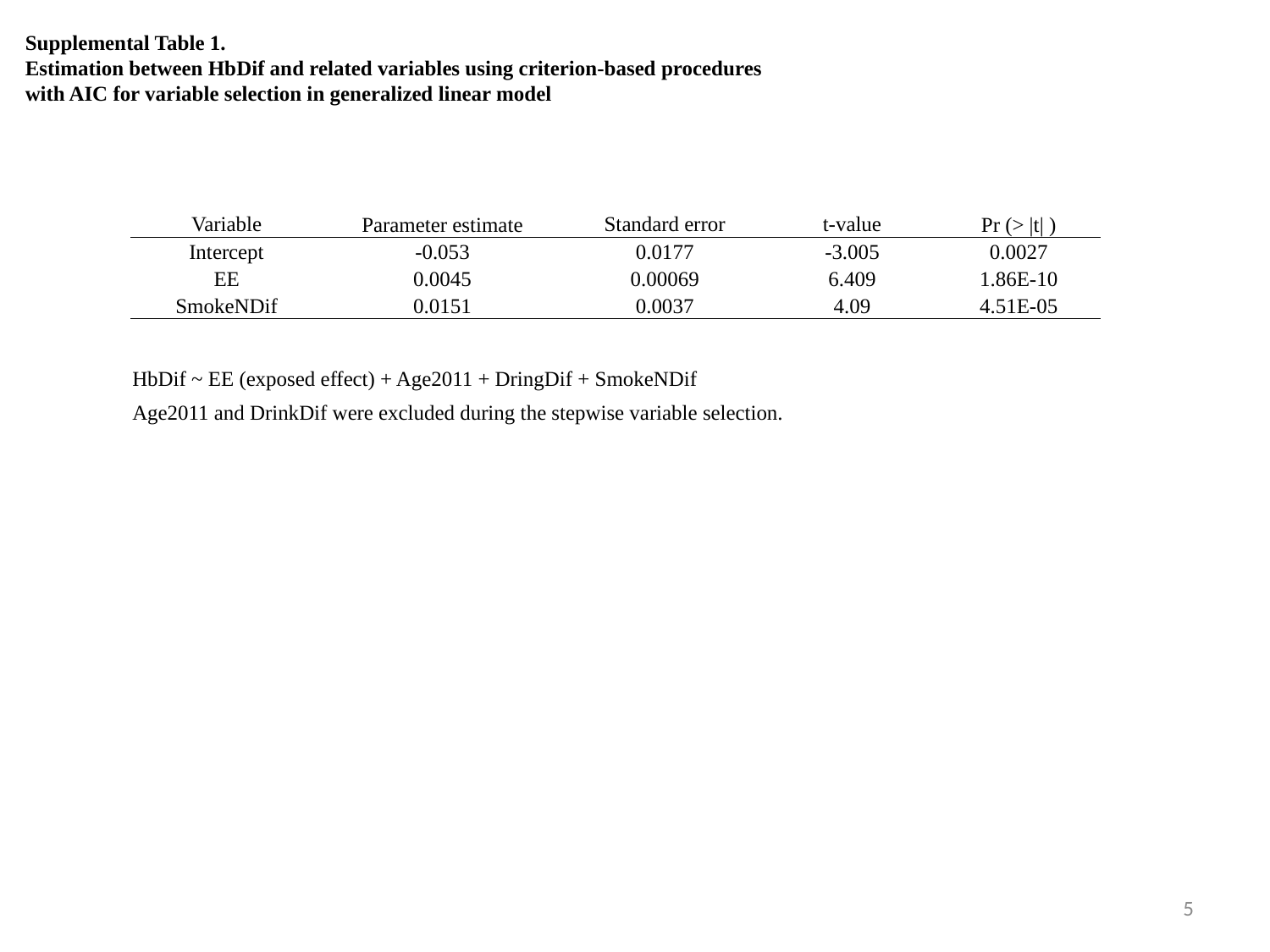

Supplemental Table 1.
Estimation between HbDif and related variables using criterion-based procedures
with AIC for variable selection in generalized linear model
| Variable | Parameter estimate | Standard error | | t-value | Pr (> |t| ) |
| --- | --- | --- | --- | --- | --- |
| Intercept | -0.053 | 0.0177 | | -3.005 | 0.0027 |
| EE | 0.0045 | 0.00069 | | 6.409 | 1.86E-10 |
| SmokeNDif | 0.0151 | 0.0037 | | 4.09 | 4.51E-05 |
| | | | | | |
| HbDif ~ EE (exposed effect) + Age2011 + DringDif + SmokeNDif | | | | | |
| Age2011 and DrinkDif were excluded during the stepwise variable selection. | | | | | |
| | | | | | |
5
